# The cross-kingdom interaction between *Helicobacter pylori* and *Candida albicans*

**DOI:** 10.1371/journal.ppat.1009515

**Published:** 2021-05-06

**Authors:** Xi Chen, Xuedong Zhou, Binyou Liao, Yujie Zhou, Lei Cheng, Biao Ren

**Affiliations:** 1 State Key Laboratory of Oral Diseases & National Clinical Research Center for Oral Diseases & West China School of Stomatology, Sichuan University, Chengdu, China; 2 Department of Operative Dentistry and Endodontics, West China Hospital of Stomatology, Sichuan University, Chengdu, China; Geisel School of Medicine at Dartmouth, UNITED STATES

## *Helicobacter pylori* infection and transmission routes

*Helicobacter pylori* is a gram-negative microaerophilic bacterium. The infection of *H*. *pylori* can increase the risk of gastric cancer which is the second leading cause of cancer death worldwide [1]. The World Health Organization’s International Agency for Research on Cancer (IARC) has classified *H*. *pylori* as a type I (definite) carcinogen since 1994. *H*. *pylori* infection is a global health problem. In developed countries, its infection rate is 20% to 50%, while in developing countries, the infection rate of middle-aged people has reached 80% [2]. The fecal–oral and the oral–oral routes are considered as the main transmission routes of *H*. *pylori* [3]. Nevertheless, only *H*. *pylori* genes have been detected in saliva and dental plaques, but culturable *H*. *pylori* has not been isolated yet in large quantities [4], indicating that there may be some new strategies of *H*. *pylori* to implement its transmission through oral cavity.

*Candida albicans*, a dimorphic fungus, is one of the most common fungi in the human body [5]. It was noteworthy that *C*. *albicans* and *H*. *pylori* were abundant in certain human niches, such as the root canal necrotic pulp, stomach, duodenum, and vagina [6], suggesting that *C*. *albicans* may interact with *H*. *pylori* to promote the growth, spread, and infection of *H*. *pylori* in some nonadaptive condition, such as the oral cavity and vagina.

### The synergy between *H*. *pylori* and *C*. *albicans* in gastric diseases

*H*. *pylori* infection is positively correlated with yeast in gastric diseases [7]. A total of 36% gastric ulcers patients, 2% non-ulcerative dyspepsia patients, and 56% large-scale gastric ulcers (greater than 2 cm) patients with *H*. *pylori* have fungal co-colonization in the upper gastrointestinal tract, such as *C*. *albicans* and *Candida krusei*, indicating the strong relationship between fungi and *H*. *pylori* in ulcerative lesions [8]. *C*. *albicans* is highly correlated with *H*. *pylori* in gastric cancer, peptic ulcer, and chronic gastritis patients. The gastric ulcer patient with *C*. *albicans* and *H*. *pylori* in the stomach developed even larger ulcers. The presence of *C*. *albicans* was closely related to the prolongation of gastric diseases by the increase of healing time and persistence of clinical symptoms [9,10]. The strong positive correlation between *C*. *albicans* and *H*. *pylori* in the development of gastric diseases indicates their synergistic pathogenesis [7]. *C*. *albicans* may enhance the colonization, toxicity, and pathogenicity of *H*. *pylori* especially in gastrointestinal diseases through adhesion and the formation of a mixed species, like that *C*. *albicans* promotes the pathogenicity of other bacteria [11].

### Potential for *H*. *pylori* residence within *C*. *albicans* vacuoles

*H*. *pylori* is an invading intracellular pathogen, and its entry into cells such as gastric epithelial cells, dendritic cells, and macrophages is one of the reasons for the failure of antibiotics treatment [12]. Interestingly, *H*. *pylori* was also found to enter *C*. *albicans* yeast cells. Moving “bacterial-like bodies” in the vacuoles of the *C*. *albicans* yeast cells from the stomach were observed, and these were identified as *H*. *pylori* by PCR and immunofluorescence [13–15]. These “invaded” *C*. *albicans* cells were able to survive from the exposure of high temperature, dryness, and antibiotics. The *H*. *pylori* in their vacuole showed an active state of motion under these conditions [13], suggesting that the internalization of *H*. *pylori* into *C*. *albicans* can protect *H*. *pylori* from strict conditions. Meanwhile, the invading *H*. *pylori* seems to be vertically transmitted to the daughter cells of *C*. *albicans* and continue to express its own proteins through its proliferation within the yeast cells [16]. The *C*. *albicans* containing *H*. *pylori* in this fast-moving state can also be observed from other body sites, such as the oral cavity and vagina [17]. The frequency of *H*. *pylori*–invaded *C*. *albicans* in the oral cavity of the normally born babies is higher than that of cesarean birth, indicating that *C*. *albicans* in the vagina may be the main reservoir for transmitting *H*. *pylori* to the newborns through their oral cavity [17]. It’s possible that *C*. *albicans* can act as a shelter and an oral transmission promoter for *H*. *pylori* [16,18,19]. Besides *C*. *albicans*, clinical isolates of *Candida dubliniensis*, *C*. *krusei*, and *Candida tropicalis* have also found the *H*. *pylori* internalization by amplifying the 16S rDNA of *H*. *pylori* [20,21], suggesting that this kind of interaction manner between *H*. *pylori* and yeast can occur in different species.

*H*. *pylori*–invaded *C*. *albicans* is not only widely distributed in the human body, but also abundantly in food, such as yogurt, grape juice, bread, preserves, fruits, and honey [22]. *C*. *albicans* may protect *H*. *pylori* against the environmental stresses in these habitats. The internalization into *C*. *albicans* could be a crucial strategy for *H*. *pylori* to survive and transmit in a variety of environments especially the nonadaptive conditions.

It’s still worthwhile to note that there are some moving volutin (polyphosphate) granules known as “dancing bodies” in the vacuoles of the *C*. *albicans* cells [23], which do not depend on the cell cycle phase, but on the growth stage, metabolic level, and stress responses [24]. The presence of *H*. *pylori* may induce a stress response that activate the formation of volutin granules in the vacuoles of *C*. *albicans*. Therefore, more evidence is needed to distinguish the “dancing bodies” in the vacuole and evaluate the cross-kingdom interaction mechanisms between *C*. *albicans* and *H*. *pylori*.

The pores of *C*. *albicans* cell wall may act as the channel which the *H*. *pylori* can pass through. The FITC-IgY-*H*. *pylori* can enter into *C*. *albicans* yeast cells through the cell wall and eventually accumulated in the vacuoles [25]. However, the specific mechanism still needs further investigation, such as the cell wall/membrane remodeling of *C*. *albicans* when cocultured with *H*. *pylori*.

The internalization of *H*. *pylori* into *C*. *albicans* is highly influenced by pH stress as the percentage of yeasts harboring bacteria at an acidic pH was nearly twice than that observed in the neutral environment [26]. But when the pH is lower than 4, the number of yeasts harbored bacteria falls sharply. This may be due to the change in the cell wall structure and the surface electric charge of *C*. *albicans* under acidic conditions, such as the significant loss of the fibrillar layer, the increased exposure of chitin and β-glucans [26], and the change of zeta potential of *C*. *albicans* [27].

### Possible relationship between the interaction of *C*. *albicans* and *H*. *pylori* with gastrointestinal flora

The clinical outcomes of the *H*. *pylori*–infected patients were quite different due to the diversity of the gastric and intestinal microbiota [28]. *H*. *pylori* infection can reduce the diversity of gastrointestinal flora. The Actinobacteria, Bacteroidetes, Firmicutes, and Proteobacteria showed a decreased abundance, while Spirochetes and Acidobacteria showed an increased abundance [29]. The eradication of *H*. *pylori* can significantly increase the abundance of the gastric and downstream intestinal flora [30], such as *Clostridium*, *Bacillus*, etc., while the abundance of fungi such as yeast was significantly increased [31]. Disorders of the intestinal microbiota from *H*. *pylori*–infected patients may lead to the destruction of the intestinal barrier, thereby increasing the susceptibility to inflammatory bowel diseases [28]. The colonization of *C*. *albicans* in the gastrointestinal tract can also influence gastrointestinal flora. *C*. *albicans* creates a niche to increase the growth and survival of various microorganisms through the formation of the poly-species biofilms with bacteria, such as *Bacteroides* spp. and *Firmicutes* spp. [32]. However, *C*. *albicans* can reduce intestinal colonization of *Clostridium difficile*, a pathogenic agent of inflammatory bowel diseases [33].

The “standard triple treatment” of *H*. *pylori* infection recommended a composition of a proton pump inhibitor plus clarithromycin, together with amoxicillin or metronidazole. However, the effectiveness of this treatment declined to unacceptably low levels due to the antibiotic resistance in less than a decade [34]. Moreover, the use of antibiotics from the “standard triple treatment” significantly reduced the alpha and beta diversity of gastrointestinal flora, mainly including *Bifidobacterium bifidum*, *Lactobacillus acidophilus*, and *Escherichia coli*. The surviving bacteria, such as *E*. *coli*, increased the resistant capability to these antibiotics [35]. The antibiotics-affected gastrointestinal flora became more vulnerable to the colonization of *C*. *albicans* [36]. *C*. *albicans* colonized in the gastrointestinal tract may protect *H*. *pylori* from antibiotics killing through endosymbiosis or biofilm formation, then disrupt gastrointestinal metabolism and immunity to increase the risk of other diseases.

### Probiotics may serve as a potential treatment

Probiotics have some natural advantages, such as safety, immunomodulation, and anti-pathogen abilities. They are usually used alone or in combination with drugs to treat gastrointestinal diseases. *Lactobacillus* spp. resided in the human stomach can inhibit *H*. *pylori* by secreting antibacterial substances, competing for binding sites, or interfering with the adhesion process to prevent *H*. *pylori* colonization, enhance the mucus barrier function, and reduce the host’s inflammatory response [37]. The triple therapy supplied with probiotic, including *Saccharomyces boulardii*, *Limosilactobacillus reuteri*, and *Lactobacillus casei*, for the treatment of *H*. *pylori* infection has the best therapeutic effect with the least adverse events [34]. Meanwhile, probiotics, such as *Lactobacillus* sp., can inhibit the adherence, biofilms, hyphae formation, and virulence expression of *C*. *albicans* [38]. *Lactobacillus rhamnosus* L34 can attenuate local inflammation, severity of intestinal leakage, fecal malnutrition, and systemic inflammation in mice infected with *C*. *albicans* [39]. Accordingly, the application of probiotics may be served as a potential treatment to inhibit the synergistic infections caused by *H*. *pylori* and *C*. *albicans*.

**Fig 1:**
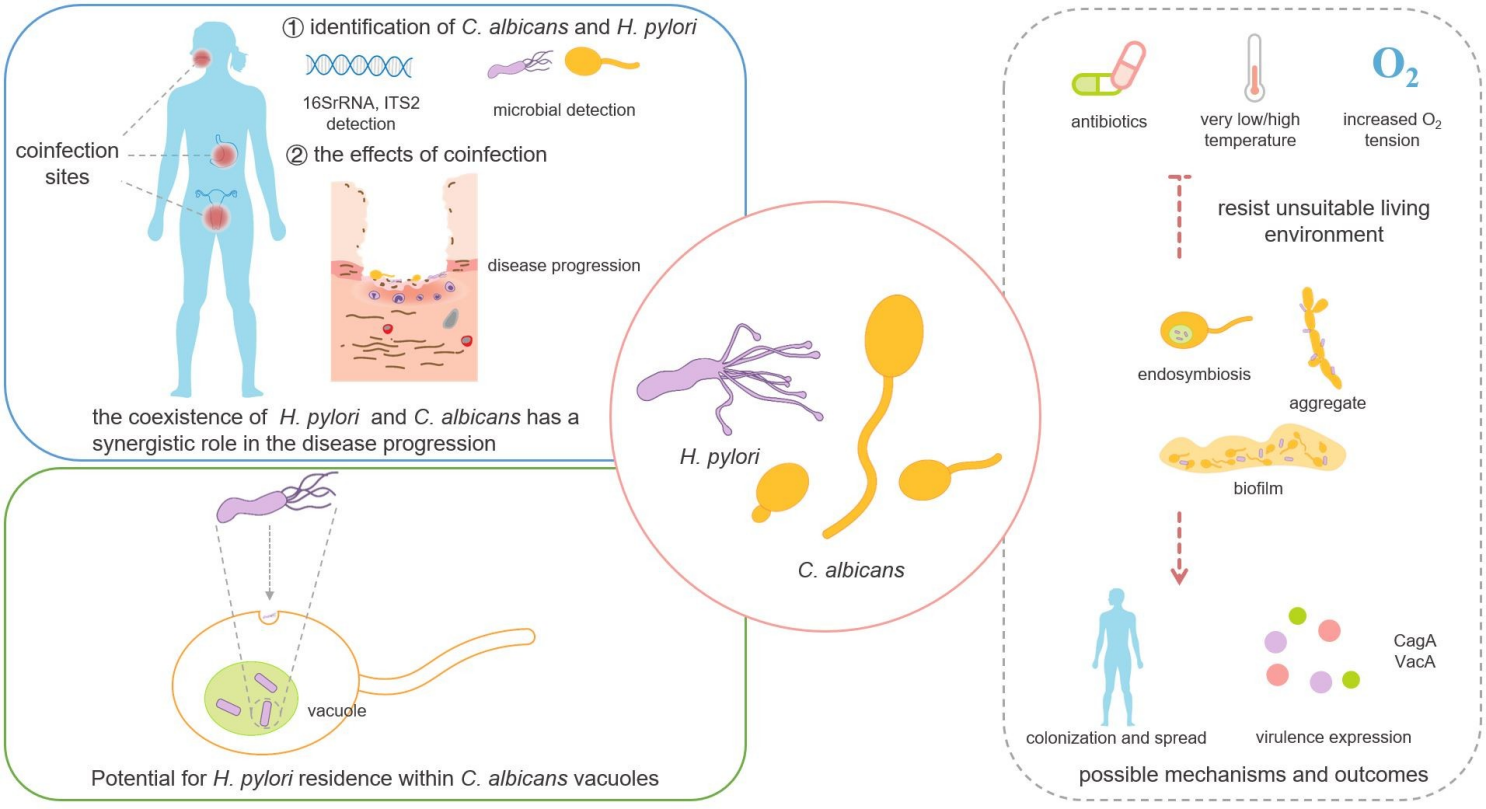
The cross-kingdom interaction between *Helicobacter pylori* and *Candida albicans* and the possible mechanisms and outcomes. There is a strong positive correlation between *H*. *pylori* and *C*. *albicans* in the colonization and synergistic pathogenesis. *H*. *pylori* potentially inhabit within *C*. *albicans* vacuoles through the endocytosis pathway. The interactions between *H*. *pylori* and *C*. *albicans* may increase the resistance to the killing effect of antibiotics and unfavorable living environment through endosymbiosis, adhesion, or formation of mixed biofilms, then promote the spread and colonization and increase the virulence factors to affect the occurrence and development of diseases.

More than 50% of people were infected with *H*. *pylori* worldwide [2]. The triple or quadruple therapy with different antibiotics in clinical has gradually failed to eradicate the *H*. *pylori* infection mainly due to the increased drug resistance of *H*. *pylori* [40]. Moreover, these antibiotics disrupted the balance of the gastrointestinal flora, metabolism, and immunity and even increased the risk of other diseases [28,36]. The investigation of the interaction between *H*. *pylori* and other microorganisms can be one of the new ways to treat *H*. *pylori* infection. *C*. *albicans* may increase the expression of virulence factors and the growth and colonization of *H*. *pylori* in different environmental conditions to promote its pathogenicity and transmission (Fig 1), since *C*. *albicans* synergized with *H*. *pylori* to resist its unsuitable living environment and increase the infection. Their cross-kingdom interactions may be a new target for the prevention, diagnosis, and treatment of *H*. *pylori* infection.
